# Continuous Snow-Cover Monitoring and Avalanche Detection with a Novel Sensor Array Box †

**DOI:** 10.3390/s26072041

**Published:** 2026-03-25

**Authors:** Markus Hoffmann, Michael Brauner, Christian Rachoy, Thomas Dolleschal, Ingrid Reiweger

**Affiliations:** 1 Hoffmann-Consult, 1230 Vienna, Austria; 2 ÖBB-Infrastruktur AG, 1020 Vienna, Austria; michael.brauner@oebb.at (M.B.);; 3 BLADESCAPE Airborne Services GmbH, 2320 Schwechat, Austria; 4 Institute of Mountain Risk Engineering, Department of Landscape, Water and Infrastructure, BOKU University of Natural Resources and Life Sciences, 1180 Vienna, Austria

**Keywords:** monitoring, avalanche slopes, event detection, systematic patterns, risk analysis

## Abstract

Snow avalanches pose a serious hazard in snow-covered, mountainous areas. In order to protect inhabited areas and infrastructure such as roads and railway lines, avalanche protection measures need to be taken. In addition to permanent, technical protection measures, temporary, organizational measures, which are based on risk assessments by local avalanche warning commissions, are utilized. These avalanche risk assessments rely on regional avalanche bulletins, weather forecasts, local expertise, and information on current snowpack conditions. Our research seeks to enhance knowledge of current snowpack and avalanche conditions by providing in situ monitoring of potential avalanche slopes. Therefore, we developed a novel sensor box array, peakr, consisting of multiple sensor units deployed by hand or by drone at key avalanche slope locations throughout the winter season. The sensors continuously measure temperature, humidity, position, and snowpack movement. Data are transmitted via LoRaWAN and GSM, stored locally, and accessed through a web platform. Automated analysis using a decision tree and event-detection algorithm triggers immediate alerts to responsible personnel via SMS and email. This paper presents an overview of the peakr sensor array and web platform, focusing on data analysis and avalanche events from the Arlberg ski resort in winter 2023/2024, supported by webcam time-lapse validation.

## 1. Introduction

Large areas in the alpine regions of Austria, as well as other countries with mountainous areas, are vulnerable to natural hazards, particularly snow avalanches in winter [1]. In order to protect inhabited areas and infrastructure such as roads and railway lines, avalanche protection measures need to be taken. Technical protection structures are one method used to safeguard exposed areas permanently. An overview of the most important technical avalanche protection works, as well as their applicability, can be found in Rudolf-Miklau et al. (2008) [1]. In cases where permanent protection measures are either not feasible or insufficient, organizational measures are taken, depending on the current avalanche risk, which is assessed by local avalanche commissions. As an example, a local avalanche commission might recommend temporarily closing a railway line [2]. The general role and work of the avalanche commissions in Austria are described by Studeregger and Renner (2019) [3]. Details on the decision-making and documentation process of the avalanche commissions of the Austrian Railways, in particular, can be found in Rachoy and Ortner (2018) [2]. The avalanche risk assessment by the avalanche commissions relies on high-altitude weather data, historic events, local insights, route-specific weather forecasts, and the regional avalanche bulletin. A key aspect of thorough risk assessment is snow observation, such as the continuous monitoring of potential avalanche slopes [2,3].

### 1.1. Avalanche Types and Monitoring Focus

Snow avalanches are categorized according to their release mechanism, namely into loose snow avalanches, slab avalanches, and glide-snow avalanches [4,5]. Loose snow avalanches start due to a loss of cohesion at the top of the snowpack, gathering more snow as they descend and forming a triangular shape. Slab avalanches form as a fracture initiates and propagates in a weak snow layer beneath a cohesive slab. Glide-snow avalanche release results from a loss of friction between the snowpack and the moist or wet ground or weak layers. As the interface between the snowpack and the (usually smooth) ground becomes increasingly wet, the friction is reduced, leading to snow gliding and often also to glide-snow avalanches. As glide-snow avalanches require liquid water to be present within the snowpack, and within the category of “wet” versus “dry” avalanches, they can therefore be characterized as “wet”. Mayer et al. (2024) [6] showed that, due to climate change, there will be an absolute increase in wet-snow avalanche days at high elevations and a relative increase in wet-snow avalanche days at low and mid elevations in the Swiss Alps until the end of the century. We expect similar results in Austria, with the exact differentiation between “high” and “low” elevations depending on site-specific properties. Moreover, glide-snow avalanches cannot, or at least very rarely (and only at high cost) [7], be triggered artificially, making their prediction particularly important [8]. The monitoring focus of this particular project is therefore on glide-snow avalanches.

### 1.2. Avalanche Monitoring Devices

Avalanche monitoring is crucial for detecting avalanches [8,9,10], building up consistent databases, providing further insights into avalanche mechanisms, trigger conditions, and avalanche rescue conditions [11,12,13,14], and supporting avalanche risk prediction [15]. According to Fox et al. (2024) [16], well-known monitoring devices can be categorized into the following types:

- Snow sensors: They directly measure parameters within the snowpack, such as snow depth, snow temperature [17], snow density [18], or mechanical forces. Examples of the latter include shear frames or force measurement devices to measure strength or stresses within the snowpack [19,20,21,22]. Values measured with these sensors might give an indication of imminent avalanche release.
- Automated weather stations: They monitor meteorological conditions relevant to snowpack stability and avalanche formation, such as precipitation, temperature, air moisture, radiation balance, and wind. These are essential input factors for snowpack modeling [23,24] and avalanche prediction [8].
- Seismic, infrasound, and acoustic sensors: Avalanches can be quickly detected by means of the ground noise they generate during initiation and descent. The sensor arrays analyze the typical sound signature, back azimuth, and the apparent velocity of the moving snow mass. This makes it possible to provide long-range, real-time detection and warnings using fiber optic sensors, seismic sensors (geophones), or infrasound arrays [25,26]. Moreover, detecting acoustic emissions within the snow yields information on cracking processes within the snowpack, potentially leading to avalanche release [27,28,29,30].
- Radar systems: Radar systems with Doppler or continuous wave technology can detect fast-moving volumes based on density differences. This enables the detection of the layering and dynamics within the avalanche mass [9,31,32]. When in line-of-sight, detection is possible at distances of up to several hundred meters in near real-time, providing early warning for medium- to large-scale avalanches. This sensor type can be used efficiently in air-based operations using drones (unmanned aerial vehicles (UAVs).
- Webcams: Cost-effective webcams provide avalanche commissions with live images, and time-lapse photos are used to monitor avalanche slopes [16,33,34]. At an experimental level, deep learning approaches can be employed to detect and segment avalanche starts, enabling near-real-time detection. This sensor type can be used efficiently in air-based operations using a UAV.
- Drone-based monitoring: This method utilizes unmanned aerial vehicles (drones) for visual inspection and mapping of avalanche-prone areas by means of various sensor arrays [35,36].
- Lidar (Light detection and ranging): Lidar measurements can provide high-resolution 3D mapping of snow-covered terrain to assess snow depth and, therefore, potential avalanche release depth. This sensor type can be used efficiently in air-based operations using an UAV to assess avalanche risk [35,37].
- Satellite (radar and imaging): This method provides radar data and satellite images for the detection and monitoring of large-scale avalanche events [38,39].
- Multi-sensor arrays for avalanche dynamics: Multi-sensor arrays are used for high-resolution, short-term observation of avalanche dynamics in artificially triggered avalanches. This involves tracking particle trajectories and conditions, thereby improving our understanding of the processes involved. Such high-resolution multi-sensor arrays can be used for short-term observation to track avalanche dynamics based on particle trajectories and local properties in artificially triggered avalanches, thereby improving avalanche models and simulations (AvaNode and AvaRange systems [40,41]).
- Novel sensor array “peakr”: High-resolution multi-sensor array for long-term continuous measurements from within the snowpack to monitor changes and track movements in the snowpack, as well as to detect avalanches and provide warnings in case of natural avalanche events in real time.

### 1.3. Snow Probing

In the field of snow probing, commonly used sensors are operated manually for snow profiling using temperature and humidity sensors, as well as snow grain-size scales for different layers. While temperature measurements are rather straightforward, the detection of liquid water in snow remains a challenge. Typical methods and devices for measuring snowpack water content are as follows:

- Snow profiling: The main method for determining snowpack properties is manual snow profiling. When performing a snow profile, one determines the different layers of the snowpack, usually distinguished by hand hardness. For each layer, one then additionally assesses grain type, grain size, and liquid water content. This is done by carefully extracting grains from each layer with a black (for optical contrast) crystal screen and examining the grains and potential liquid water with a magnifying glass. A detailed description of snowpack properties can be found in The International Classification for Seasonal Snow in the Ground by Fierz et al. (2009) [42]. Snow temperature is measured with a thermometer, which is inserted into the snowpack at 10 cm vertical intervals. Snow density is measured by carefully punching out (without compacting) a representative volume of the snowpack or a snow layer with a cylinder of a known volume. This volume is then weighed, and the density is calculated. Snowpack stability is determined by manual stability tests, such as the compression test, the shovel test, or the extended column test [43]. Manual profiling is done regularly by avalanche commissions, avalanche observers, and the avalanche warning services in Austria. However, as manual snow profiling requires people to be on site, it is not possible on avalanche slopes at times of increased risk. A detailed description of snow profiling, the associated snowpack parameters, and observation techniques can be found in the avalanche classics by McClung and Schaerer (2006) [44] and Tremper (2018) [45].
- Snow moisture measurement plates: Snow moisture measurement plates measure the volumetric liquid water content of snow via its dielectric properties. This measurement system was first developed by Denoth (1994) [46] and further developed by Wolfsperger et al. (2023) [47]. A drawback of this method is that the dry-snow density needs to be known as input to be able to calculate snow wetness from the dielectric properties. This issue can be solved quite well, however, by using an estimated snow density derived from grain shape and hardness or by measuring snow density manually with a conventional snow cylinder. As far as potential avalanche slopes are concerned, however, a bigger drawback is that, again, people are required at the measurement site, so it is not possible on avalanche slopes at increased risk.
- Time-domain reflectometry: Schneebeli et al. (1998) [48] adapted sensors commonly used for measuring soil moisture to measure density and wetness within snow. The physical principle is time-domain reflectometry (TDR), where the time of an electromagnetic pulse sent along a cable and returned is measured. The dielectric properties of the surrounding material affect the signal’s travel time, allowing estimation of snow density or liquid water content. As far as we know, while widely used in the soil community, this method has not established itself for measuring moisture within snow. Moreover, it might again be hard to retrieve data from avalanche slopes.
- Global positioning system signal attenuation: Koch et al. (2019) [49] used the attenuation of signals from global positioning system (GPS) devices to measure snow wetness. While this method is very promising, it measures the snow water equivalent and liquid water content over the whole height of the snowpack, rather than within a specific layer.
- Ground-penetrating radar: Schmid et al. (2015) [50] developed a ground-penetrating radar (upGPR) in combination with GPS to provide non-invasive measurements of snow stratigraphy as well as snow liquid water content. While this is a very promising method, it requires a radar system to be buried and installed in the potential avalanche slope before winter. Moreover, the radar needs a power supply, so there needs to be at least a solar panel nearby, with the cables to the radar being buried so that they are not carried away by an avalanche.

As manual periodic measurements are limited on critical avalanche slopes, there is ongoing research on short-term monitoring of particle movements and snowpack parameters under artificial triggering conditions (e.g., [40,51]). In addition, time-lapse and continuous video monitoring have become an important tool for both validating avalanche events and improving forecasting accuracy. Over the past decade, several studies have demonstrated the effectiveness of using webcams to track snow cover evolution, glide cracks, and avalanche activity [8,25,36,52]. However, these methods do not provide continuous measurements from within the snowpack and are thus often combined with snowpack simulations and periodic manual surveys of the avalanche slopes, together with weather station data.

### 1.4. Goal and Scope of the Research

The aim of our research was to develop an autonomous sensor system to measure snowpack parameters directly within avalanche slopes and to transmit the resulting data directly to avalanche commissions to aid them in their decision-making concerning avalanche risk. The measurement data had to be transmitted wirelessly through the snow cover for analysis. Furthermore, a robust algorithm for event detection was needed to allow timely decision-making by avalanche commissions. The solution had to be field-tested on several sites for robustness and applicability. As avalanche slopes are not accessible during times of high avalanche risk, direct snowpack measurements from within avalanche slopes provide a new and very promising tool for risk assessment. This project was initiated by the Austrian railway infrastructure operator ÖBB-Infrastruktur AG, with avalanche risk assessment along railway lines in mind. The avalanches we focused on were glide-snow avalanches, which are most relevant at the moment for the ÖBB, as they cannot, or at least very rarely and at high cost [7], be triggered artificially, and will presumably stay relevant in the future [6]. The present paper provides an overview of the current state of development of our sensor array, our online data platform, as well as several observed and detected events validated using webcam time-lapse images. This article is a revised and thoroughly expanded version of a conference contribution entitled “Towards systematic patterns and avalanche risk analysis with continuous monitoring and event detection on avalanche slopes with a novel sensor array box in Austria” [53], which was presented at the International Snow Science Workshop ISSW in Tromsø, Norway, 23–29 September 2024.

## 2. Materials and Methods

### 2.1. Development of Sensor Array “Peakr”

**Figure 1:**
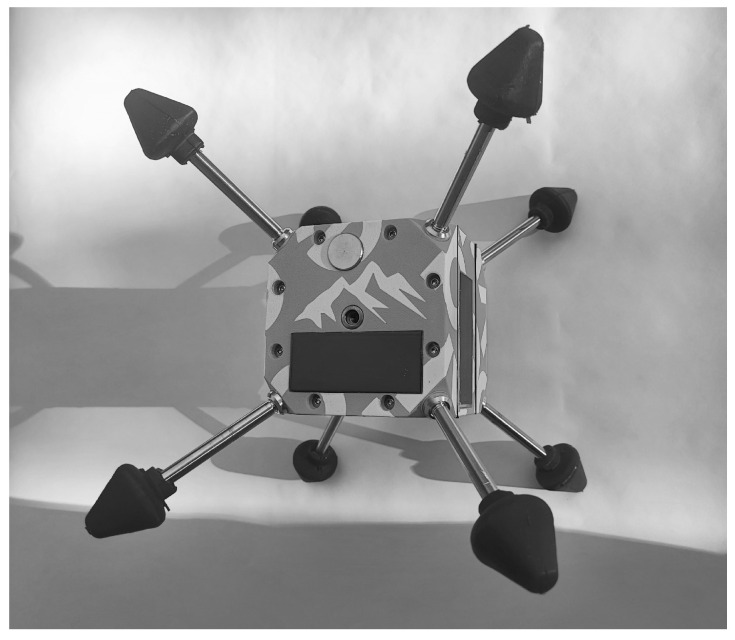
The sensor array box “peakr” containing multiple sensors, with slip guards and soft tips.

To meet the requirements of robustness, redundancy, continuous measurements, data transmission without external energy supply, as well as convenient deployment by hand or by drone, the developed solution was subject to an iterative development process. The latest version of the sensor array, Figure 1, consists of a small box (10 × 10 × 10 cm) with beveled, reinforced corners into which various types of slip guards can be screwed. The sensor array is waterproof and weighs around 1 kg, including the slip guard with soft tips, minimizing accident risks. It allows continuous, multiple, independent measurements of position and acceleration (2×), temperature, and moisture (3×) for real-time monitoring, with the data sent through the snow cover via LoRa to a gateway over distances up to a few kilometers.

The goal of multiple measurements of each parameter was to obtain reliable data and allow for robust detection of outliers or sensor failures based on independent, redundant measurements. The data are also stored locally on an SD card and can be accessed via a USB-C adapter, which also allows charging of the LiPo battery cells for redeployment. Due to initial problems with energy consumption, the sensor array and measurement interval were optimized for the battery to last for at least six months. In order to achieve these results, we specifically selected the components with the least possible power consumption, tested all deployed devices for one month at temperatures around the freezing point, and specifically tested for energy leaks based on power consumption and heat development measurements.

A specific challenge is the non-linear discharge behavior of the batteries, requiring specific testing strategies and battery management. As the GPS consumes a considerable amount of energy, the position of the device is checked once per day. The measurement interval of all sensors was set to 10 min, which has been sufficient for long-term observations and the goals of our research. Furthermore, the measurement interval can be changed remotely to any other interval, and in case of sudden changes in acceleration, the sensor system wakes up and provides additional measurements. To handle multiple devices, the sensor array can be identified based on an RFID chip, with the identification transmitted directly to the server upon power-up. To retrieve the devices after the winter period, tracking by GPS signal, last known location, as well as an acoustic signal are available. The list of the main components of the sensor array is as follows:

- STM32L0 32-bit microcontroller (ultra-low-power Arm Cortex-M0+), by STMicroelectronics (Plan-les-Ouates, Geneva, Switzerland), connected to all sensors.
- Moisture sensors are designed using the capacitive measurement principle. The moisture sensors were manifactured by Texas Instruments (Dallas, TX, USA).
- Temperature sensors—Programmable Resolution 1-Wire Digital Thermometer DS18B20, manufactured by Maxim Integrated (San Jose, CA, USA).
- Acceleration sensors from ST Microelectronics LIS3DH—MEMS digital-output motion sensor, manufactured by STMicroelectronics (Plan-les-Ouates, Geneva, Switzerland).
- GPS module—Standard MULTI-MODE SATELLITE MODULE—E108-GN Series, manufactured by Chengdu Ebyte Electronic Technology Co., Ltd. (Chengdu, China).
- LoRa Transceiver Module—Fully integrated PCB SX1276 transceiver module from Semtech (Camarillo, CA, USA).

### 2.2. Web Platform “Peakr”

**Figure 2:**
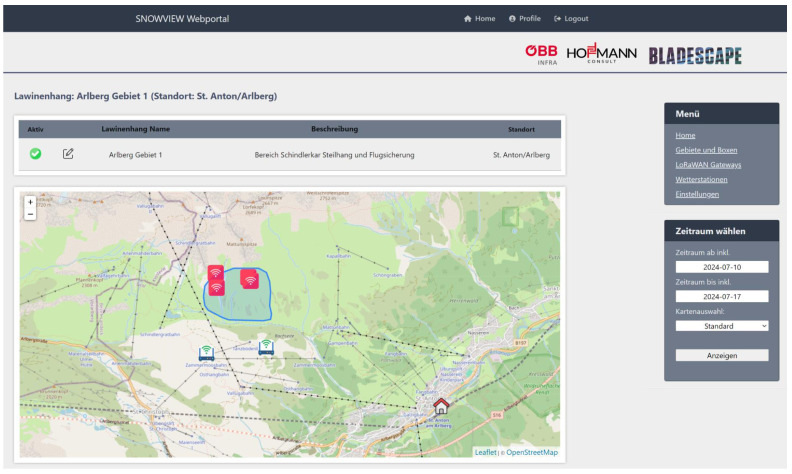
Web platform with locations of devices (pink boxes) and gateways (blue/green symbols). The data can be downloaded directly from this web platform.

The web view interface is shown in Figure 2. Our web platform requires user credentials to access the monitored areas and deployed devices of specific owners. It allows the handling of all owned devices based on bidirectional communication (e.g., firmware updates, operational parameters, measurement interval), distinguishing between the different operating modes—testing, deployment, operation, and retrieval—which is necessary to distinguish between deliberate movements and actual events.

**Figure 3:**
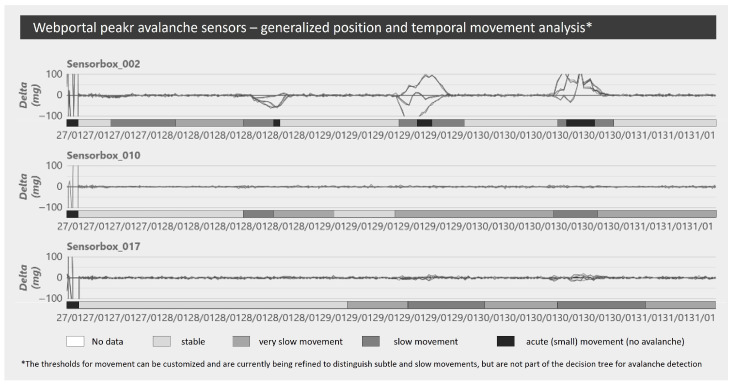
Web platform with an aggregated overview of subtle temporal movements.

The web platform provides an overview of the monitored areas as well as a list of deployed devices for direct access to the data. The monitored areas, the gateways, and deployed devices are displayed on a customizable map. Furthermore, the data from all devices can be summarized for selected sensor arrays over any selected period, highlighting subtle movements (Figure 3), temperature changes, and humidity levels with configurable thresholds, allowing comparison of monitoring data from different sensor arrays and identification of critical time frames for in-depth analysis.

For a more detailed data analysis, individual sensor arrays can be accessed from a list of active sensors, providing a direct link to the position on the map as well as the measurement data for the selected time period and a download option for raw data. The status row provides key information on the date of deployment, the latest retrieved data, and the change in position. The available data are organized in tabs, distinguishing between position, movement, temperature, humidity, change in humidity, transmitted signal strength, and the raw data table for the last measurements in a given time frame. Furthermore, there is a status section for each sensor array showing signal strength and battery state, with additional options to customize the measurement interval or update settings and firmware. For documentation and communication, it is also possible to upload photos and provide comments from observations for each specific sensor array. The field tests have shown that these options are increasingly important for large-scale observations with many sensor arrays or the management of multiple avalanche slopes.

### 2.3. Decision Tree and Event Detection

**Figure 4:**
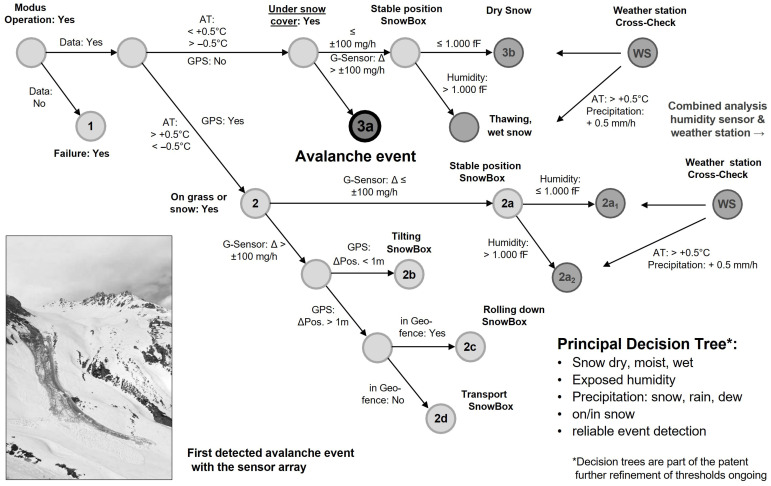
Decision tree for event detection and warning.

**Figure 5:**
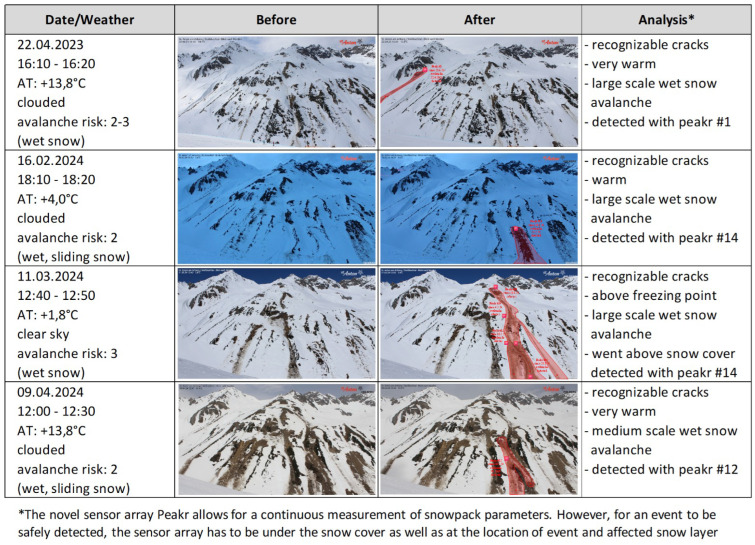
Selection of detected avalanche events with a sensor array in St. Anton/Arlberg (2023/2024).

In order to automatically detect avalanche events from the data transmitted by our sensor arrays, we developed a decision tree depicted in Figure 4. The decision tree uses a temperature threshold of ±0.5 °C to detect wet-snow conditions. No or only a very weak GPS (global positioning system) signal indicates that the sensor array is still positioned within the snow cover. An acceleration of ±100 mg/h indicates quick movement. “Quick” here means faster than slow movement due to snow gliding. All thresholds are based on periodic measurements with independent devices from snow profiles, the continuous monitoring data from the sensor arrays during the development phase [54], and our own expert judgment. The combination of a temperature of approximately 0 °C, no GPS signal, and “sudden” movement gives the outcome “avalanche event”. Our decision tree allows for automated data analysis and has so far reliably detected all glide-snow avalanche events within our observation period (Figure 5).

The decision tree was specifically designed for event classification and avalanche detection. In summary, the decision tree uses sensor fusion (temperature, acceleration, humidity, GPS availability) combined with contextual information (snow cover status, weather station data) to distinguish between avalanche events and other movement patterns. The key innovation is the differentiation between surface movements (manual handling, rolling, tilting) and subsurface movements beneath the snow cover, where avalanche events are most likely to occur.

## 3. Application and Data Analysis

### 3.1. Selected Areas for Field Tests

For the field tests and validation in the winter periods of 2022/2023 and 2023/2024, typical areas with regular avalanche occurrence adjacent to train tracks (Mallnitz, Lermoos), in ski resorts (Axamer Lizum, Hafelekar, St. Anton/Arlberg), or with a stable snow cover for validation measurements (Niederalpl, Schneeberg) in Austria were chosen.

### 3.2. Overview of Avalanche Events and Characteristics

For field tests and system validation, we chose the test site with the highest number of glide-snow avalanches, which is located in the area of the ski resort St. Anton/Arlberg, Austria. The monitored slope is a large slope that is well-known for snow gliding and glide-snow avalanches at elevations between 1800 and 2500 m a.s.l. (Steißbachtal). The ski resort provided extensive support in the deployment of the sensor arrays, as well as its expertise. Together with these local experts and through a thorough analysis of webcam data, 91 events could be identified in the area, allowing an independent validation of detected events from the sensor arrays. For the given area, 54 events in the season 2022/2023 and 37 events in the season 2023/2024 (total = 91 events) could be identified based on webcam data. Figure 5 provides insights into four medium to large events that were both recorded on webcam and independently detected by the deployed sensor arrays using the decision tree.

**Figure 6:**
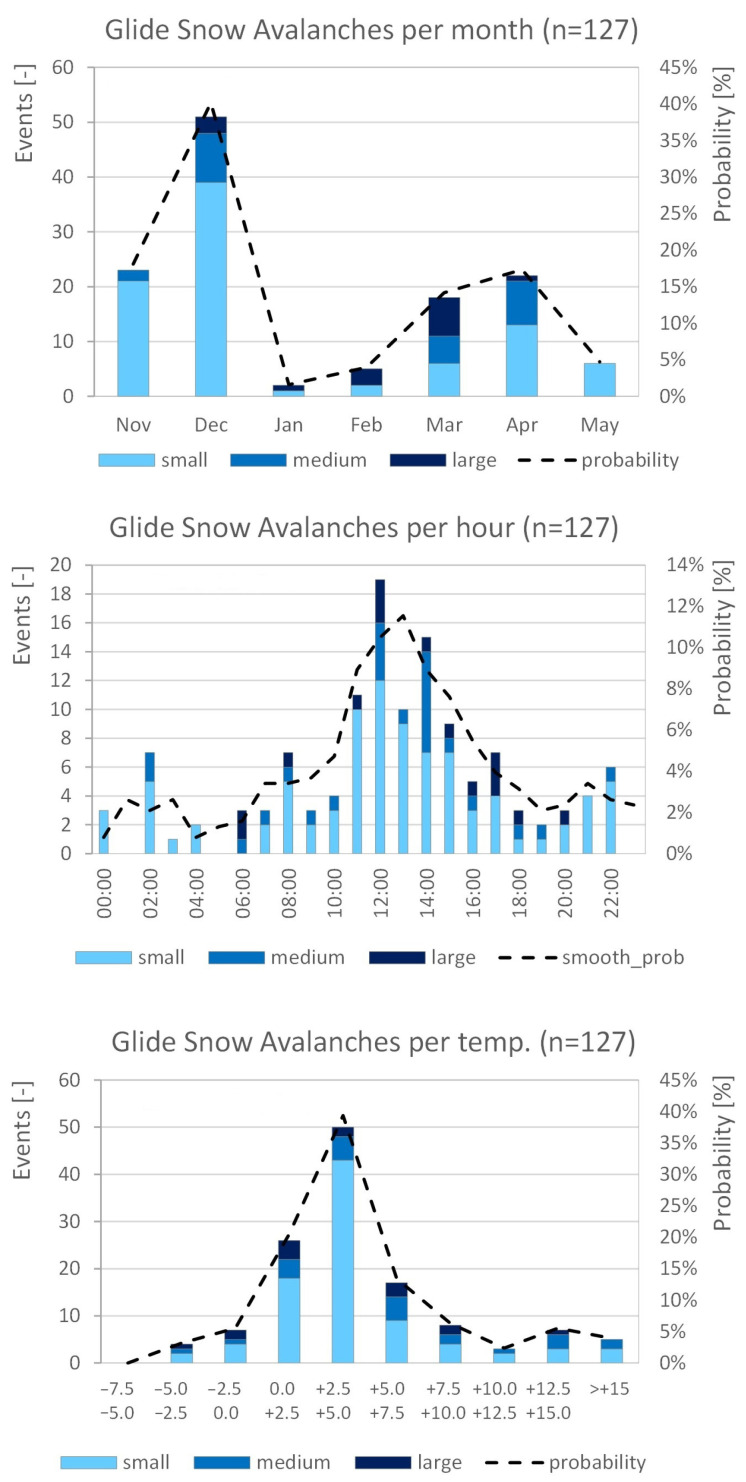
Seasonal distribution, diurnal distribution, and distribution by temperature of observed glide-snow avalanche events in all field test areas (*n* = 127). The **uppermost** plot shows the number of glide-snow avalanche events observed in each month (seasonal distribution). The **middle** plot shows the number of glide-snow avalanche events by hour of day (diurnal distribution), and the **bottom** plot shows the distribution of observed glide-snow avalanche events by air temperature.

Based on a systematic analysis of the field test sites at St. Anton/Arlberg (Winter 2022/2023 and 2023/2024) and all other test sites in Winter 2023/2024 from webcam images, manual observations, and monitoring by our peakr system, the glide-snow events were analyzed based on their occurrence and occurrence probability by month, by time of day, and ambient temperature (Figure 6). According to the analysis of this observation period, the first period with increased glide-snow avalanche risk was roughly from the 20th of November to the 20th of December, and the second lasted about two months from the 15th of February to the 15th of April. Moreover, the majority of events occurred between 11:00 and 17:00 (60%) and at ambient temperatures of 0.0 °C to 7.5 °C (73%). With temperatures above 0.0 °C as well as heat radiation from the sun on clear days, mainly between 10:00 and 16:00, a thawing process is induced in the snow-cover. As the temperatures get warmer from February to April, the average humidity in the snow cover and, thus, the glide-snow avalanche risk increase. Apart from the physical mechanism, these processes can be observed by combining the continuous moisture measurements with data from adjacent weather stations. In order to establish a quantitative relation between the risk increase and increasing temperatures and moisture levels in the snow cover, further observations over several winter seasons, as well as calibration of the sensor arrays, are needed.

### 3.3. Event Analysis St. Anton/Arlberg

**Figure 7:**
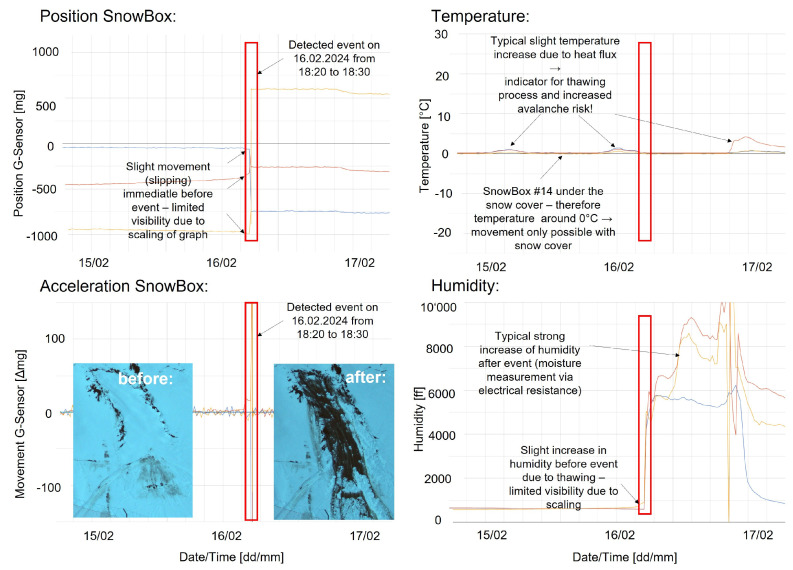
Measurements of sensor array #14 before, during, and after the glide-snow avalanche event in St. Anton/Arlberg on 16 February 2024. The two left plots show the position and acceleration measurements of all three acceleration sensors. The upper right plot shows the measurements of the three temperature sensors, and the lower right plot shows the measurements of the three humidity sensors. In each graph, the glide-snow avalanche event is marked with a red box.

As an example, the measurement data of the detected event on 16 February 2024 between 18:10 and 18:20 are shown in Figure 7. From the position data, a slight movement immediately before the event can be observed (upper left). The actual event is characterized by a sudden acceleration of the G-Sensor (lower left). As the sensor array was under the snow cover, the temperature was almost constant around 0.0 °C with a periodic slight increase in the temperature of the sensors on the upper side of the sensor array towards the snow surface. The moisture measurements suddenly increased immediately during and after the event, which is to be expected due to the capacitive measurement principle and increasing pressure, as well as the morphological changes during the event. These results prove the feasibility of reliably monitoring avalanche slopes and detecting events with the developed sensor arrays and online platform, while also indicating possibilities for quantitative risk assessment, systematic deployment, and the future identification of risk factors to support avalanche commissions in their risk assessment.

### 3.4. Avalanche Frequency and Risk Mapping

The systematic monitoring and analysis of glide-snow avalanches over several winter periods on critical slopes may also provide further insights into avalanche risk, possible areas for deployment of sensor arrays, as well as data for avalanche risk modeling and mapping. As an example, the results of the avalanche event analysis in St. Anton/Arlberg are presented for the winter periods of 2022/2023 and 2023/2024, together with the deployment locations for 2023/2024 and the georeferenced mapping of annotated and detected avalanches based on webcam time-lapse images. Initially, this time-consuming task was performed manually based on sensor array data analysis and comparison of webcam images.

**Figure 8:**
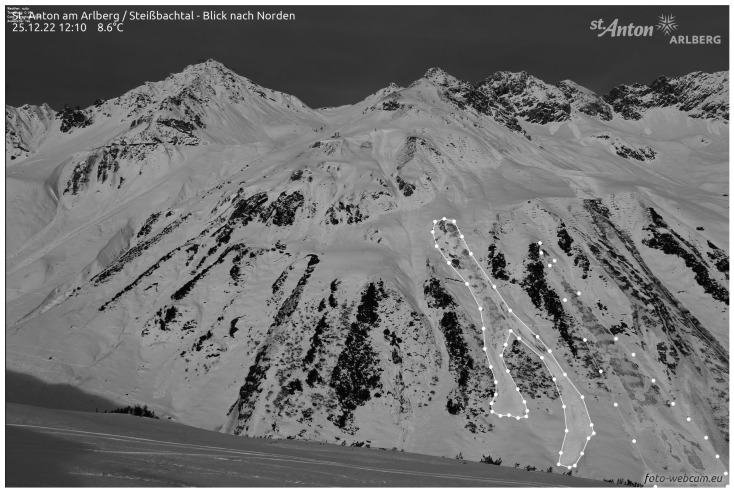
Avalanche annotation and detection in webcam image pairs (before/after) with an experimental Python tool (Phython version 3.8.10).

**Figure 9:**
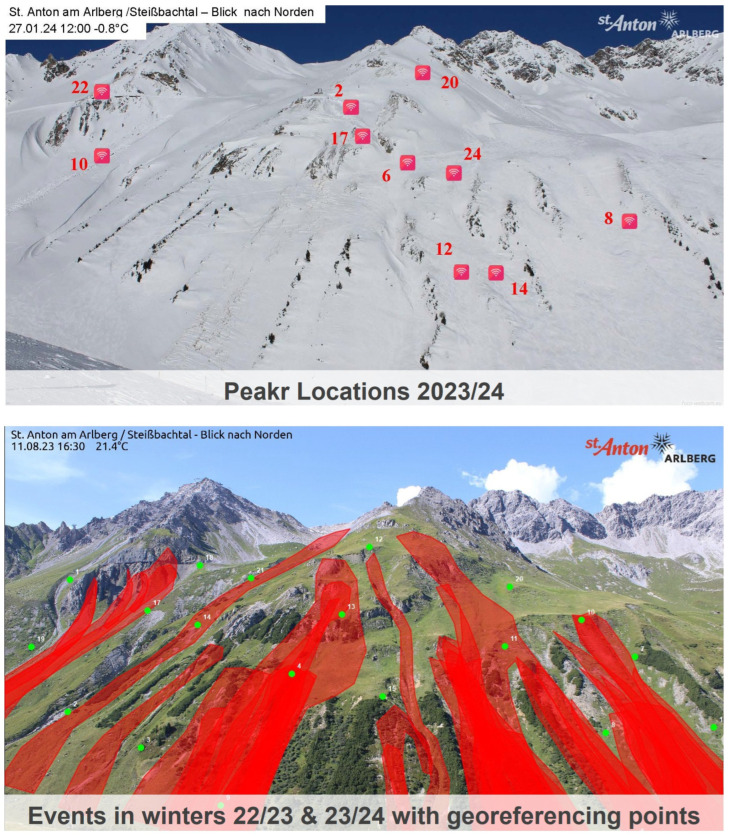
The upper image shows the monitored glide-snow avalanche slope in St. Anton/Arlberg in January 2024. The locations of the peakr array boxes are marked by red squares with the number of the respective peakr array box written next to it. The lower image shows the same glide-snow avalanche slope in August 2026. All observed glide-snow avalanche events from the winters of 2022/2023 and 2023/2024 are mapped on the image (red). The green dots are the georeferencing points.

For a more systematic analysis, an experimental Python tool was developed (Figure 8 and Figure 9) to detect glide-snow avalanches from images automatically downloaded from webcam data. The avalanche detection algorithm includes image augmentation (e.g., increasing image contrast, detecting edges and contours, and increasing texture sensitivity), annotation of image pairs before and after events, and a training and detection pipeline based on UNET, DeeplabV3, and SiameseDifference models. To achieve high detection rates, the key was found to be the use of image pairs taken at short time intervals, allowing the models to focus on pattern changes related to avalanches. The detection can be performed in batch mode for analysis or in live mode, allowing the webcam to be used as a cost-efficient additional tool together with the developed sensor arrays, except in adverse weather conditions with limited visibility. For systematic analysis of detected events from webcam images, a small database was built. Based on this, it is now possible to analyze all events of one or several winter periods on an avalanche slope. In the experimental tool, a map interface was developed, allowing users to load a reference image of the avalanche slope. This reference image, as a frontal view of the slope with the annotated and detected avalanches, is then transformed onto the map based on reference points on both the reference image and the map, using a transformation matrix and the 3D thin plate spline method, or 3D-TPS (Thin Plate Spline Method). Based on systematic monitoring as well as manual or computer vision-based automated webcam analysis for several winter periods together with slope properties, semi-automated mapping of avalanche frequency and avalanche risk is considered feasible, based on the results obtained with the developed experimental tool. Thus, the mapping of several avalanche events over a longer time period using heat and risk mapping is feasible. At the time of writing, the risk-mapping functionality is still in development based on the following concepts, but observations over a longer period of 5 to 10 years are also needed to account for differences in winter weather conditions:

- A general avalanche risk map for optimal sensor deployment from a multi-year avalanche frequency analysis. The risk map shows where avalanches are most likely to occur, created by analyzing several years of avalanche events. The map works like a heatmap (hot colors = higher risk), without assuming that the avalanche patterns necessarily stay the same over time.
- A time-dependent risk map considering factors from snowpack monitoring, slope characteristics, weather data and prediction, as well as recent events on the avalanche slope from the database.

The image classification and mapping are independent of each other but are used in a complementary manner. In most current camera systems, images are taken every 10 to 30 min. The main goal of the image classification is to determine the location, extent, and path of avalanches and provide the basis for a wider coverage in visible areas at times with good visibility. Furthermore, image classification can be used for avalanche frequency analysis and risk mapping, enabling evidence-based placement of sensor systems. However, the cameras do not provide physical continuous measurements from within the snow cover or immediate warnings in case of events.

## 4. Discussion

### 4.1. Power Consumption and Robustness of the Sensor Boxes

One of the key challenges in the development of the autonomous sensor array box was to provide continuous measurements during an entire winter season without interruption. Our first designs fell short, as the battery was depleted after 2–3 months. In order to achieve a more compact design for deployment by drone and longer battery life, all components were specifically selected for low energy consumption, and each component was tested individually for power consumption, isolation, and leakage, and underwent endurance testing at temperatures below the freezing point. In summary, this helped us to achieve a verified battery life of 5–7 months. For sustainability, the batteries can be recharged, ensuring continued use for several seasons.

The GPS is the sensor with the highest power consumption and is thus checked only once per day to ensure battery endurance. The main use of the GPS is to confirm location after deployment and to support retrieval, as in the intended use case. In the snow cover, no GPS signal can be received, and all movements in the snow are tracked by the acceleration sensors. However, it is possible to change the GPS checking frequency remotely with admin rights from the application.

Our observations on sensor robustness are based on both the design and evidence from field observations. All relevant sensors are redundant (temperature, humidity, and acceleration are each measured three times), with all data saved locally to an SD card (secure digital memory card) in case data transmission is interrupted. All sensor array boxes could be retrieved so far (*n* = 30), and in some very rare gaps in data transmission, the missing data could be retrieved later directly from the boxes. Moreover, in the four cases where sensor array boxes were swept away by avalanches, the boxes survived.

### 4.2. Avalanche Event Detection

The movement data of a sensor array can be tracked based on GPS (surface position only) and using a G-Sensor, but this alone is not sufficient to reliably detect an event, as the device can be moved manually, can tilt during thawing periods or slow movements of the snow cover, or roll down on the snow surface (states 2a to 2d in Figure 4). However, if the sensor array is under the snow cover at a depth of 0.5,m or more, typically no GPS signal is available, and the three temperature sensors provide independent continuous measurements from −0.5 °C to +0.5 °C (states 3a and 3b in Figure 4). We have not observed any false positive avalanche events so far.

So far, the decision tree for detecting glide-snow avalanche events from sensor-box data is based solely on whether the sensor is positioned within wet snow (temperature of ±0.5 °C and no GPS signal) and “fast” (as opposed to “slow” from snow gliding) movement of the sensor box via the acceleration sensors. The moisture sensors are included in the sensor box for planned future applications, namely to assess preconditions for glide-snow avalanche release (e.g., a moist to wet interface between the snowpack and the ground) and to develop not only a detection system but also an early warning system for glide-snow avalanches.

The detection of avalanches with image classification methods is complementary to the developed sensor system in several ways. For areas where camera images are available, they provide a wider observation range and can capture events in locations where no sensors are placed. However, due to the limits of the line of sight, or in areas with no camera available, as well as during bad weather, the sensor system provides continuous data from within the snow cover on the development of its physical properties, as well as on slight movements that are not visible in the camera data, and provides immediate warning in case of events. Furthermore, both the image classification and sensor systems allow independent detection and validation of events for analysis.

The GPS signal is mainly used during deployment and placement, and for retrieval. Furthermore, as the GPS signal after placement is only activated once per day to save battery, and its accuracy is limited, it is a weak indicator for movements below a scale of a few meters. Furthermore, under the intended conditions within the snow cover, there is no signal. However, if the temperature sensors show temperatures outside of the range of S = 1 (below −0.5 °C, above +0.5 °C) and a GPS signal is available, we can conclude that the sensor is not within the snow cover, and thus no alarm is triggered, because any movement can be either rolling down or due to an avalanche event. This can be validated either by camera images, if available, or by using more than one sensor system on the same slope. The decision tree reflects this (state 2 in Figure 4).

### 4.3. Recommended Sensor Placement and Monitoring Strategies

The placement strategy of the sensor array boxes depends on the avalanche risk at the time of placement. Accordingly, the sensors are placed manually by the avalanche commissions in the most critical areas of avalanche slopes based on local experience, as long as the avalanche risk is low. During times with high avalanche risk or if manual placement is infeasible, the sensor system can be deployed and dropped by our drones at a specified GPS location, with the placement being verified by the camera of the drone. The developed avalanche frequency and risk mapping will allow a quantitative validation of local experience for optimal distribution and placement of the sensors on avalanche slopes.

The data from the sensor array can be combined with weather stations, webcam analysis, and a snowpack model in a consistent database. This combination of comprehensive data can be used for systematic avalanche risk analysis and the identification of avalanche risk factors. A larger and continuously updated data set will also allow us to update and refine the decision tree. Periodic updates of the decision tree will ensure general applicability and reliable event detection, as the number of events in the deployment area was relatively small (four events in St. Anton, and one event in Lermoos).

Spatial avalanche risk maps, such as the one shown in Figure 9, yield valuable information on where to place the peakr sensor array boxes, namely in the areas most prone to avalanche release. In cases where such maps are not available, sensors are best placed based on the expert judgment of local avalanche commission members.

The development of efficient monitoring strategies using the sensor array and the web platform largely depends on the monitoring goals and expected results, the application area, the specific topographic situation, as well as the avalanche frequency. In order to achieve good results with the least possible effort, the following procedure based on field-test experience is recommended:

(a) First comes the definition of the kind of events one wants to monitor; in our case, these were glide-snow avalanches.
(b) Then the monitoring area needs to be analyzed, and the following questions answered: What are the potential avalanche slopes? What is the (roughly) expected avalanche frequency (e.g., almost daily to once a season)? What is our deployment strategy? Do we want to place the sensors permanently for the whole season or on short notice in case of increased avalanche risk?
(c) The equipment is prepared accordingly (number of sensor array boxes, LoRa-gateways, use of drones), and the users are trained to use the equipment.
(d) The duration of the monitoring phase is decided based on interest (e.g., the road to be protected is only open for a certain period of time) and financial or personnel restrictions. Do we want to monitor for either an entire winter season or a designated time frame with a high risk of e.g., glide-snow avalanches (e.g., end of January to the end of April)?
(e) The final phase consists of result evaluation, event validation, and potential for improvement, with reports and dissemination. For the preparation of risk maps, long-term monitoring and systematic data collection are needed.

### 4.4. Planned Research and Development

Combining our newly developed sensor arrays and the experimental Python tool (Phython version 3.8.10) with webcams is considered feasible for a cost-efficient avalanche monitoring solution. This will also be a sound basis for developing the Python tool to integrate further sources of information into an avalanche database, allowing the calculation of avalanche frequency maps and improving risk assessment. In summary, such a system may offer a comprehensive, cost-effective monitoring approach for avalanche slopes and support decision-making in the future. Future developments and research by the project team will include the following:

- Refinement of the decision tree and event detection algorithms based on more extensive field data.
- Integration with other data sources, such as weather stations and snowpack models, for more comprehensive avalanche event and risk analysis, not only for glide-snow avalanches but also for other avalanche types.
- Adaptation of the sensor array to measure ground temperature and humidity on the slope surface to detect thawing processes at the boundary layer between soil and snow.
- Development of avalanche frequency and risk maps based on long-term monitoring data with automated analysis and mapping in the experimental Python tool.
- Improvement of machine learning techniques for the reliable detection of avalanches from webcam images, as well as the identification of subtle patterns in snowpack conditions that may indicate increased avalanche risk.
- Expansion of the system to cover more avalanche-prone areas, potentially creating a network of monitored slopes and a comprehensive avalanche event database.
- Further miniaturization and cost reduction of the sensor array to allow for more widespread deployment with small commercial-grade drones, also limiting the costs of lost or non-reusable sensor arrays.
- Investigation of the integration of additional sensors, weather, and slope parameters, as well as snowpack simulations that could enable an automated avalanche risk prediction.
- Improvement of the user-friendly interfaces and decision support tools of the online platform for avalanche commissions and other stakeholders, also including versions in different languages.
- Building a comprehensive database on observed and validated avalanche events for model validation and avalanche risk prediction with semi-automated mapping and risk analysis.

## 5. Conclusions

This paper presents a novel approach for avalanche monitoring and risk assessment. We developed a sensor array and web platform called “peakr,” allowing continuous monitoring of avalanche slopes. Deployed manually or by drone, our sensor arrays provide real-time data on temperature, humidity, position, and movement. The sensor array is a small, robust device that measures temperature, humidity, position, and movement under the snow cover and can survive avalanche events. The sensor array has a default measurement interval of 10 min that can be changed remotely, and it wakes up in case of sudden acceleration, providing additional measurements. The thorough testing and specific battery management enable continuous monitoring for up to six months. At low avalanche risk, our sensor boxes can be deployed manually. At high avalanche risk, we developed a deployment system using drones, with the drone operator standing at a safe location far enough away from the avalanche slope. Our sensor array transmits data wirelessly through the snow cover to a database and web platform. The web platform provides real-time data analysis and visualization, with automated event detection and risk assessment. For further robustness in case of transmission errors, the data are stored on an SD card in the device. More details on the development process of our system can be found in [54]. Field tests were conducted at various locations in Austria, with a focus on the ski resort St. Anton/Arlberg in the Steissbachtal area due to frequent avalanche events. Despite limited snowfall in recent winters, 127 glide-snow avalanche events in our testing regions based on webcam images were analyzed together with data from weather stations in the seasons 2022/2023 and 2023/2024. In the specific areas in St. Anton where our sensor arrays had been deployed, all four avalanche events were detected with the decision tree and independently validated. The paper presents the statistical analysis of all recorded events as well as a table of the four detected events and sample data from one selected event, demonstrating the system’s ability to detect glide-snow avalanches and provide insights into snowpack conditions before, during, and after events. From the perspective of the authors, there are several highlights, ranging from the development of a robust sensor array and deployment by drone to the detection and validation of several avalanche events with the decision tree.

## 6. Patents

The sensor array called “peakr” and the algorithms on the platform were patented: Patent A50911/2023, Branding AM10329/2025.

## Data Availability

The sensor array data from the events and the time-lapse imagery and statistical analysis of events from this study will be made available upon reasonable request to the corresponding author. Due to privacy agreements with funding and partner institutions (ÖBB-Infrastruktur AG and ski resort operators), some data may be anonymized.
